# Pathology of the Blood in Inebriates

**Published:** 1848-08

**Authors:** 


*Pathology of the blood in Inebriates.* By T. J. W. Pray, M. D., of Boston.

Sir: I have been requested, by some of my medical friends in Boston, to collect the facts relating to the state of the blood taken from an inebriate in South Berwick, Me., and transmit them to your Journal.

It is well known to the members of the medical profession, that liquids, and even mineral and vegetable substances, taken into the stomach, are shortly found in different parts of the system. Madder, for instance, when internally administered, imparts a like color to the milk and urine, and to the bones of animals, without materially affecting the healthy action of any tissue, or sensibly deranging the constituent parts of the solids or fluids, unless too long persevered in. Like results, varying according to the qualities of the fluids, are found, whenever any fluid is taken into the stomach capable of absorbtion. But there are very few known cases in which so much alcohol has been absorbed into the system as to change the chemical qualities of the blood, or so modify it, that its watery proportions should give place to the fluid which has been immoderately indulged in. The case which we shall now briefly refer to, plainly shows that such may be the fact.

A Mr. Thompson, aged 35 years, had long been subject to fits of intoxication, and was daily accustomed to the demands of a ruling passion. For five days previous to the examination of the blood, he had been in a beastly state of inebriation; and, indeed, it was found, upon inquiry, that he had drank, in that time, *two gallons* of "West India Rum," At the expiration of the fifth day, he went to Dr. J. C. Hanson, complaining of the usual symptoms of drunkenness, and wishing medical aid. Dr. H., seeing that he did not require any active medical treatment, but rather the *expectant* plan, concluded to deplete him a very little, for an experiment. The blood was forthwith drawn; and it was found destitute, in a measure, of its watery elements—alcohol having been substituted therefor. Immediately a lighted taper was applied to it, and it began to burn with a flame similar to that of alcohol. This produced such an effect upon the inebriate, that he refrained from his intemperate habits, and afterwards became a more sober man.

The fact, that the blood did burn, can be substantiated by the testimony of Drs. Jewett and Hanson, and other respectable citizens of South Berwick, who were eye-witnesses at the time the blood was drawn, and saw the experiment tried.—*Boston Med. and Surg. Journal.*

Dover, N. H., May 15, 1848.

